# Structural Disadvantage in Adolescence and Biological Aging in Early Midlife

**DOI:** 10.1001/jamanetworkopen.2026.11913

**Published:** 2026-05-11

**Authors:** Taylor W. Hargrove, Alena Sorensen D’Alessio, Sylvie Tuder, Ariayana Harrell, Lauren Gaydosh, Audrey L. Kelly, Allison E. Aiello, Kathleen Mullan Harris, Brandt Levitt, Chantel L. Martin

**Affiliations:** 1 Department of Sociology, University of Maryland, College Park; 2 Maryland Population Research Center, University of Maryland, College Park; 3 Southern Population Aging Research Center, University of Maryland, College Park; 4 Department of Epidemiology, Gillings School of Global Public Health, University of North Carolina at Chapel Hill, Chapel Hill; 5 Carolina Population Center, University of North Carolina at Chapel Hill, Chapel Hill; 6 Department of Sociology, University of North Carolina at Chapel Hill, Chapel Hill; 7 Department of Epidemiology, Columbia Aging Center, Mailman School of Public Health, Columbia University, New York, New York

## Abstract

**Importance:**

Upstream social determinants, including structural disadvantages, are critical drivers of health and aging. While structural disadvantages shape biological aging and inflammatory processes among older adults, it is less clear how this association emerges and endures over the life course.

**Objective:**

To assess whether adolescent exposure to structural disadvantage is associated with epigenetic aging and inflammation-related DNA methylation (DNAm) in early midlife and to evaluate whether associations differ by race.

**Design, Setting, and Participants:**

This prospective cohort study used data from non-Hispanic Black and White respondents in the National Longitudinal Study of Adolescent to Adult Health (Add Health). Add Health comprises a nationally representative cohort of US adolescents in grades 7 to 12 in 1994 (wave I) followed for over 20 years across 6 waves of data. Venous blood samples were collected (2016-2018) and analyzed for DNAm (2021-2024) among Add Health respondents in early midlife (ages 33-43 years at blood collections). Data were analyzed from September 2024 to February 2026.

**Exposure:**

The main exposure was structural disadvantage in adolescence, assessed as 5 county-level economic, education, and segregation indicators from the 1990 decennial US Census.

**Main Outcomes and Measures:**

The main outcomes included 3 epigenetic clocks (PhenoAge, GrimAge2, and DunedinPACE) and 2 measures of inflammation-related DNAm (C-reactive protein [CRP] and tumor necrosis factor-α). Confirmatory factor analysis was used to derive a latent factor of structural disadvantage in adolescence, and multivariate regression models assessed the association between the structural disadvantage latent measure and each outcome.

**Results:**

Data from 3788 participants (mean [SD] age at wave V, 38.4 [0.01] years; 50.9% [SE, 1.1%] female and 49.1% [SE, 1.1%] male; and 19.7% [SE, 0.9%] Black and 80.3% [SE, 0.9%] White) were analyzed. Considering average associations across the sample, exposure to higher vs lower levels of structural disadvantage in adolescence was associated with accelerated epigenetic aging (GrimAge2: β, 0.35 [95% CI, 0.09-0.61]; DunedinPACE: β, 0.08 [95% CI, 0.03-0.13]) and greater CRP-related DNAm (β, 0.07 [95% CI, 0.02-0.12]), even after adjusting for self-reported race and family socioeconomic status. Findings from an interaction model suggested that while Black respondents experienced faster epigenetic aging and greater CRP-related DNAm overall, the association between adolescent structural disadvantage and these outcomes was slightly negative for Black respondents, yet positive for White respondents.

**Conclusions and Relevance:**

In this prospective cohort study of adults in early midlife, the results suggest that early-life contexts were important factors for accelerated epigenetic aging and CRP-related DNAm. These findings enhance understanding of when and how disparities in aging-related diseases may emerge, informing effective solutions for addressing the rising burden of aging-related diseases.

## Key Points

**Question:**

Is adolescent exposure to structural disadvantage associated with epigenetic aging and inflammation-related DNA methylation (DNAm) among Black and White adults in early midlife?

**Findings:**

In this cohort study of 3788 Add Health respondents, greater exposure to structural disadvantage in adolescence was associated with accelerated epigenetic aging and greater C-reactive protein (CRP)–related DNAm. These associations were slightly negative for Black respondents, yet positive for White respondents.

**Meaning:**

These findings suggest that early-life contexts are important factors for accelerated epigenetic aging and CRP-related DNAm, and they elucidate when and how disparities in aging-related diseases may emerge.

## Teaser

This cohort study explores whether exposure to structural disadvantage in adolescence is associated with epigenetic aging and inflammation-related DNA methylation among Black and White adults in early midlife.

## Introduction

The aging population in the US is growing rapidly. While life expectancy has increased over the past 50 years, the prevalence and burden of aging-related diseases is rising.^1,2^ Black individuals in the US experience a disproportionate burden of aging-related diseases, including Alzheimer disease and related dementias, heart disease, diabetes, chronic inflammation, and advanced physiological aging, relative to their White counterparts.^3,4,5,6,7^ With the population older than 65 years expected to increase by approximately 30% in the next 25 years,^8,9^ it is imperative to understand the multifaceted drivers of disparities in aging-related disease risk, particularly by race and prior to old age.

Existing evidence suggests that structural racism is a critical driver of aging-related disease outcomes and inequalities.^10,11^ Structural racism involves the interconnection of institutions, laws, policies, practices, and ideologies that organize racialized groups into a hierarchical structure and distribute valued resources according to that structure.^12,13^ Structural racism patterns racialized disparities in health and aging by shaping unequal exposure to health-relevant risks and resources across the life course.^11,14^

Prior work has documented associations between structural racism and various aging-related outcomes, including cardiovascular and metabolic morbidity, inflammation, cognition, and epigenetic aging^14,15,16,17,18^; yet several limitations remain. First, previous research generally has examined exposures to structural racism in adulthood or associations between racism, socioeconomic status, and epigenetic aging in children and adolescents.^18,19,20,21^ While important, structural racism is a dynamic, life-course exposure. Less attention has been given to the role of early-life contexts in shaping later-life epigenetic aging. Early life stages such as childhood or adolescence are sensitive periods during which exposures may have particularly strong, lasting effects on subsequent health and aging via the accumulation and activation of risks and resources.^22,23,24^

Second, few studies have empirically assessed how exposures like structural racism shape biological function to influence aging-related diseases. Ecosocial theory posits that social structures and exposures become embodied at a biological level via biopsychosocial responses.^25^ Increases in the incorporation of DNA methylation (DNAm) data with social surveys have facilitated testing of these pathways. DNAm is an epigenetic process that contributes to gene expression and is sensitive to environmental factors. Using array-based technology and machine learning approaches, composite measures of DNAm, also known as *DNAm surrogates*, are trained to predict aging-related phenotypes. Epigenetic clocks are one such surrogate developed as molecular measures of biological aging—first to predict chronological age,^26,27^ and more recently trained on age-related biomarkers^27,28,29^—and their rate of change.^30^ Epigenetic age acceleration occurs when DNAm age exceeds chronological age, and this acceleration is associated with numerous aging-related disease and mortality outcomes.^31^ Other DNAm surrogates for specific inflammatory markers, such as C-reactive protein (CRP), serve as accurate indicators of inflammation-related phenomena over and above the measured blood proteins themselves.^32,33^

While prior work has linked social exposures to epigenetic aging and inflammation-related DNAm,^34,35^ few studies have examined associations between dimensions of structural racism and these outcomes.^36,37^ One particularly important dimension of structural racism is racism-related structural economic and social disadvantage (RR-SESD). RR-SESD captures the racially patterned allocation of educational, economic, and residential opportunities rooted in historical and contemporary forms of discrimination (eg, redlining, restrictive covenants, and legal and institutional barriers to education and employment).^14,38^ Consequently, RR-SESD shapes life chances and environments (eg, education and employment opportunities, political disenfranchisement, social networks, and exposure to pollutants and toxins) that directly and indirectly influence aging-related outcomes via stress and inflammatory processes.^39,40^ No studies, to our knowledge, have examined whether early-life exposure to RR-SESD is associated with epigenetic aging and inflammation-related DNAm in early midlife, before symptoms of many aging-related diseases emerge.

Moreover, the extent to which such associations differ for Black and White adults is unclear. In the US, structural racism has historically and contemporaneously constrained Black individuals’ access to educational and economic opportunities and residential contexts. Acquiring comparable levels of resources and opportunities may therefore not translate into comparable lived experiences, exposures, or health-protective benefits across racial groups.^41,42,43^ These racially patterned constraints and exposures to unique race-related stressors may become biologically embedded through pathways linked to chronic stress and inflammation. Accordingly, we used data from a national cohort of US adults who have been followed up for over 20 years to address the following questions: (1) To what extent is adolescent exposure to RR-SESD associated with epigenetic aging and inflammation-related DNAm in early midlife? (2) Do these associations vary by race?

## Methods

This cohort study, which used nationally representative longitudinal data from the National Longitudinal Study of Adolescent to Adult Health (Add Health), was approved by the University of North Carolina at Chapel Hill Institutional Review Board. Add Health participants provided informed consent. We followed the Strengthening the Reporting of Observational Studies in Epidemiology (STROBE) reporting guideline.

### Sample

In the Add Health study, an initial cohort of 20 745 adolescents (aged 12-20 years) drawn from school rosters was sampled in 1994 (wave I) and followed up for more than 20 years across 6 waves of data.^44^ We used data from wave I (1994) and wave V (2016-2018). A subsample of wave V respondents (n = 5381 of 12 300) participated in an in-home biomarker data collection, which included a venous blood draw for 93.1% of participants (ages 33-43 years at the time of blood draw). Valid DNA samples were collected, and DNAm was assayed for 4621 of these participants. Our study sample included self-reported non-Hispanic Black and White respondents (n = 761 excluded) with valid wave V DNAm data who were not pregnant at wave V (n = 52 excluded) and had nonmissing county residence at wave I (n = 30 excluded). We limited our sample to Black and White respondents because our study examines structural disadvantages rooted in anti-Black racism in the US context. The specific dimensions of structural disadvantages we assessed (eg, economic and educational disparities, as well as segregation) reflect racialized systems that have disadvantaged Black individuals relative to White individuals. Our final analytic sample comprised 3788 respondents. We compared the total wave V epigenetic sample to our analytic samples on key covariates, finding that the exclusion criteria did not notably bias the analytic sample (eTable 1 in Supplement 1).

### Measures

#### Epigenetic Clocks

Outcomes included 3 epigenetic clocks, all batch corrected.^45^ Methylation of 850 000 CpG sites was measured using the Infinium Methylation EPIC BeadChip (Illumina Inc).^46^ More information about the DNAm processing in the Add Health study is available elsewhere.^35^ Epigenetic age was estimated using GrimAge, version 2 (hereinafter, GrimAge2)^47^ and PhenoAge,^31^ 2 epigenetic clocks trained to predict morbidity and mortality. Epigenetic age estimates were calculated using published algorithms for each clock. Residuals from regressing each age estimate on chronological age (current year minus birth year) were used as outcomes. Positive residuals (epigenetic age greater than expected chronological age) indicate elevated epigenetic age compared with others of the same chronological age.

Rate of epigenetic aging was estimated using DunedinPACE,^30^ which identifies the rate of physiological decline for every 12 months of calendar time in the years prior to sample collection. Standardized DunedinPACE rates in units of SDs were examined. Positive *z* scores (respondent epigenetic aging rate greater than mean epigenetic aging rate) indicated physiologic decline occurring at a rate faster than was expected due to passing calendar time compared with others of the same chronological age.

#### Inflammation Surrogates

We examined DNAm surrogates for CRP^48^ and circulating tumor necrosis factor (TNF)-α,^49^ computed as weighted sums of CpGs identified in epigenome-wide association studies. The eMethods in Supplement 1 present detailed descriptions of each outcome.

#### Racism-Related Structural Economic and Social Disadvantage

We operationalized the RR-SESD measure with 5 county-level indicators from the 1990 decennial US Census, which were merged to wave I residences: (1) proportion of residents with income below the 1989 federal poverty level; (2) proportion of residents with a bachelor degree or more; (3) proportion of adult residents who were unemployed; (4) proportion of residents who identified as Black; and (5) proportion of county youths aged 4 to 19 years living with a mother who did not graduate from high school, who was divorced or separated, and who had income below the 1989 poverty level. While we use the term *disadvantage* to describe this index, not all indicators—specifically, proportion of Black residents—represent inherent disadvantages. We included the proportion of Black residents as a proxy for racial residential segregation, which results from long-standing forms of exclusion of Black individuals from housing, lending, and financial markets in the US.^38^ Such exclusions have created conditions in which higher-proportion Black neighborhoods are more likely to experience concentrated socioeconomic disadvantages and harmful ecological environments.^50^

Following previous studies using a theoretically informed approach to measuring aspects of structural racism,^14^ we conducted a county-level confirmatory factor analysis to derive a latent factor score reflecting exposure to RR-SESD at wave I (n = 267 unique counties). All 5 county-level measurement variables were standardized and loaded onto a single latent variable. Maximum likelihood estimation with robust (Huber-White) SEs was used. Factor scores for all counties were estimated using the latent model and then assigned to each respondent based on wave I residence. Higher values of the latent construct indicated that respondents were living in counties with more families with incomes below the poverty line, fewer residents with high educational attainment, more residents who were unemployed, more residents who were Black, and more youths who were living with a mother with fewer socioeconomic resources. Additional detail on the latent model, including a path diagram with factor loadings, is available in the eMethods in Supplement 1.

### Statistical Analysis

Two ordinary least-squares regression models were estimated for each outcome, with the county-level RR-SESD factor score as the primary exposure. Model 1 adjusted for potential confounders of the association, including respondent age at wave V, sex (female [reference] or male), parental income (in thousands; continuous) and educational attainment (less than college or college degree or more [reference]; dichotomous) at wave I, years in current residence at wave I (to account for duration of residence in the county; continuous), respondent self-reported race (non-Hispanic Black or non-Hispanic White), and cell type. Model 2 added an interaction term between respondent race and the RR-SESD factor to model 1.

Multiple imputation with chained equations was conducted to account for missingness in the confounder variables (eTable 2 in Supplement 1 provides more detail on missingness). Fifty imputed datasets were utilized for all descriptive statistics and model results. Descriptive statistics and models were also weighted and clustered appropriately using Add Health’s built-in nationally representative sampling weights for the wave V biomarker subsample. These weights account for Add Health’s complex longitudinal survey design and unequal probability of selection and attrition over time.^51^

Two-tailed *P* < .05 was considered statistically significant. All analyses were conducted in RStudio, version 1.3.1056 (Posit). Data were analyzed from September 2024 to February 2026.

## Results

Table 1 displays weighted descriptive statistics for all study variables, overall and by race, for the 3788 participants in our analytic sample (mean [SD] age at wave V, 38.4 [0.01] years; 50.9% [SE, 1.1%] female and 49.1% [SE, 1.1%] male; and 19.7% [SE, 0.9%] Black and 80.3% [SE, 0.9%] White). Black respondents, on average, lived in counties with greater exposure to the RR-SESD latent factor than White respondents. Additionally, Black respondents, on average, had parents with lower levels of income and education, spent fewer years in their current wave I residence, experienced faster epigenetic aging (GrimAge2 and DunedinPACE), and had greater CRP-related DNAm. Black and White respondents had similar mean values of PhenoAge, and Black respondents had lower average values of TNF-α–related DNAm.

**Table 1.** Weighted Descriptive Statistics of Study Variables for the Add Health Cohort, Total and by Race

Variable	All respondents (N = 3788)	Black respondents (n = 872)	White respondents (n = 2916)	Bivariate differences by race, β (95% CI)	*P* value
Epigenetic clock, mean (SE), y					
PhenoAge	30.1 (0.1)	30.1 (0.3)	30.1 (0.1)	−0.42 (−1.08 to 0.24)	.21
GrimAge2	48.2 (0.1)	49.8 (0.3)	47.8 (0.1)	1.73 (1.18-2.29)	<.001
DunedinPACE	1.0 (0.003)	1.1 (0.01)	1.0 (0.003)	0.61 (0.50-0.73)	<.001
Inflammation surrogate, mean (SE), *z* score					
CRP	0.03 (0)	0.04 (0)	0.03 (0)	0.69 (0.57-0.81)	<.001
TNF-α	−0.003 (0)	−0.01 (0)	−0.002 (0)	−0.38 (−0.52 to −0.24)	<.001
RR-SESD latent factor, mean (SE)	−0.2 (0.02)	0.3 (0.05)	−0.3 (0.02)	0.45 (0.36-0.54)	<.001
Age at wave V, mean (SE), y	38.4 (0.01)	38.7 (0.1)	38.3 (0.05)	0.39 (0.16-0.62)	<.001
Sex, % (SE)					
Female	50.9 (1.1)	49.9 (2.6)	51.1 (1.3)	−47.0 (−27.3 to 18.0)	.69
Male	49.1 (1.1)	50.1 (2.6)	48.9 (1.3)		
Race, % (SE)					
Black	19.7 (0.9)	NA	NA	NA	NA
White	80.3 (0.9)	[Reference]	[Reference]	[Reference]	
Parental income, mean (SE), $1000s	47.1 (1.2)	31.9 (1.8)	50.8 (1.4)	−18.93 (−23.04 to −14.82)	<.001
Parent educational ttainment, % (SE)					
Less than college	62.1 (1.1)	70.9 (2.2)	60.0 (1.2)	48.47 (25.29-71.65)	<.001
College or more	37.9 (1.1)	29.1 (2.2)	40.0 (1.2)		
Years in residence, mean (SE)	7.3 (0.1)	5.9 (0.3)	7.6 (0.1)	−1.79 (−2.41 to −1.17)	<.001

Abbreviations: CRP, C-reactive protein; NA, not applicable; RR-SESD, racism-related structural economic and social disadvantage; TNF, tumor necrosis factor.

Table 2 and Table 3 summarize the results from the regression analyses for the epigenetic clocks and DNAm surrogates for inflammation, respectively. In both analyses, the results from model 1 indicated a positive association between the RR-SESD latent factor and GrimAge2 (β, 0.35 [95% CI, 0.09-0.61]), DunedinPACE (β, 0.08 [95% CI, 0.03-0.13]), and CRP-related DNAm (β, 0.07 [95% CI, 0.02-0.12]), suggesting that greater exposure to RR-SESD in adolescence was associated with accelerated epigenetic aging and greater CRP-related DNAm relative to those with less exposure to RR-SESD. To contextualize these findings, individuals who lived in counties at the third quartile of the RR-SESD latent factor distribution (value of 0.33) during adolescence exhibited approximately 0.45 years (95% CI, 0.20-0.71), or 165 days, of additional epigenetic aging acceleration (GrimAge2) compared with demographically similar individuals who lived in counties at the first quartile of the latent factor distribution (value of −0.77). This difference amounts to approximately 9 years of accelerated aging across a 20-year span. For DunedinPACE, the difference in exposure to RR-SESD was associated with approximately 5.18 days (95% CI, 2.78-7.52 days) faster aging per chronological year.

**Table 2.** Multivariate Models of Associations Between the RR-SESD Latent Factor and Epigenetic Clocks^a^

Variable	Epigenetic clock											
	PhenoAge				GrimAge2				DunedinPACE			
	Model 1^b^	*P* value	Model 2^c^	*P* value	Model 1	*P* value	Model 2	*P* value	Model 1	*P* value	Model 2	*P* value
RR-SESD latent factor	0.15 (−0.11 to 0.41)	.24	0.12 (−0.16 to 0.41)	.40	0.35 (0.09 to 0.61)	.01	0.47 (0.17 to 0.78)	<.001	0.08 (0.03 to 0.13)	<.001	0.12 (0.06 to 0.17)	<.001
Race												
Black	−0.69 (−1.34 to −0.05)	.04	−0.71 (−1.36 to −0.05)	.03	1.23 (0.66 to 1.81)	<.001	1.30 (0.72 to 1.87)	<.001	0.48 (0.36 to 0.60)	<.001	0.50 (0.38 to 0.62)	<.001
White	[Reference]	NA	[Reference]	NA	[Reference]	NA	[Reference]	NA	[Reference]	NA	[Reference]	NA
RR-SESD latent factor × race	NA	NA	0.12 (−0.51 to 0.75)	.71	NA	NA	−0.49 (−1.06 to 0.08)	.09	NA	NA	−0.13 (−0.25 to −0.02)	.02
Age	−0.01 (−0.14 to 0.12)	.85	−0.01 (−0.14 to 0.12)	.85	−0.14 (−0.26 to −0.03)	.02	−0.14 (−0.26 to −0.03)	.02	0.04 (0.02 to 0.06)	<.001	0.04 (0.02 to 0.06)	<.001
Sex												
Female	[Reference]	NA	[Reference]	NA	[Reference]	NA	[Reference]	NA	[Reference]	NA	[Reference]	NA
Male	−0.61 (−1.08 to −0.15)	.01	−0.61 (−1.08 to −0.14)	.01	0.71 (0.28 to 1.14)	<.001	0.71 (0.28 to 1.14)	<.001	−0.14 (−0.22 to −0.05)	<.001	−0.14 (−0.22 to −0.05)	<.001
Parental income	−0.01 (−0.01 to 0)	.02	−0.01 (−0.01 to 0)	.02	−0.01 (−0.01 to 0)	<.001	−0.01 (−0.01 to 0)	.01	0	<.001	0	<.001
Parent educational attainment												
Less than college	1.04 (0.55 to 1.54)	<.001	1.05 (0.55 to 1.55)	<.001	2.21 (1.77 to 2.65)	<.001	2.19 (1.74 to 2.63)	<.001	0.41 (0.32 to 0.50)	<.001	0.40 (0.32 to 0.49)	<.001
College or more	[Reference]	NA	[Reference]	NA	[Reference]	NA	[Reference]	NA	[Reference]	NA	[Reference]	NA
Years in residence	−0.03 (−0.07 to 0.01)	.17	−0.03 (−0.07 to 0.01)	.16	−0.04 (−0.08 to 0.03)	.03	−0.04 (−0.08 to 0)	.03	−0.01 (−0.01 to 0)	.07	−0.01 (−0.01 to 0)	.08

Abbreviations: NA, not applicable; RR-SESD, racism-related structural economic and social disadvantage.

^a^ Unless indicated otherwise, values are reported as β (95% CI). All models control for batch and cell type.

^b^ Model 1 examines the association between the RR-SESD latent factor and the outcome, adjusting for covariates.

^c^ Model 2 adds an interaction between the RR-SESD latent factor and race to model 1.

**Table 3.** Multivariate Models of Associations Between the RR-SESD Latent Factor and Inflammation-Related DNA Methylation^a^

Variable	Inflammation surrogate							
	CRP				TNF-α			
	Model 1^b^	*P* value	Model 2^c^	*P* value	Model 1	*P* value	Model 2	*P* value
RR-SESD latent factor	0.07 (0.02 to 0.12)	<.001	0.11 (0.05 to 0.16)	<.001	0.03 (−0.02 to 0.09)	.28	0.05 (−0.01 to 0.10)	.10
Race								
Black	0.55 (0.43 to 0.68)	<.001	0.57 (0.44 to 0.70)	<.001	−0.41 (−0.55 to −0.27)	<.001	−0.40 (−0.54 to −0.26)	<.001
White	[Reference]	NA	[Reference]	NA	[Reference]	NA	[Reference]	NA
RR-SESD latent factor × race	NA	NA	−0.14 (−0.25 to −0.02)	.02	NA	NA	−0.07 (−0.22 to 0.09)	.40
Age	0.05 (0.03 to 0.08)	<.001	0.05 (0.03 to 0.08)	<.001	0.02 (−0.01 to 0.04)	.24	0.02 (−0.01 to 0.04)	.24
Sex								
Female	[Reference]	NA	[Reference]	NA	[Reference]	NA	[Reference]	NA
Male	−0.20 (−0.29 to −0.11)	<.001	−0.20 (−0.28 to −0.11)	<.001	−0.10 (−0.19 to 0)	.05	−0.10 (−0.19 to 0)	.04
Parental income	0	.01	0	.01	0	.70	0	.73
Parent educational attainment								
Less than college	0.33 (0.24 to 0.42)	<.001	0.32 (0.23 to 0.42)	<.001	0.03 (−0.07 to 0.12)	.60	0.02 (−0.07 to 0.12)	.65
College or more	[Reference]	NA	[Reference]	NA	[Reference]	NA	[Reference]	NA
Years in residence	−0.01 (−0.02 to 0)	.04	−0.01 (−0.02 to 0)	.05	0 (−0.01 to 0.01)	.75	0 (−0.01 to 0.01)	.72

Abbreviations: CRP, C-reactive protein; NA, not applicable; RR-SESD, racism-related structural economic and social disadvantage; TNF, tumor necrosis factor.

^a^ Unless indicated otherwise, values are reported as β (95% CI). All models control for batch and cell type.

^b^ Model 1 examines the association between the RR-SESD latent factor and the outcome, adjusting for covariates.

^c^ Model 2 adds an interaction between the RR-SESD latent factor and race to model 1.

Model 1 further indicated a significant disparity between Black and White respondents for GrimAge2 (β, 1.23 [95% CI, 0.66-1.81]), DunedinPACE (β, 0.48 [95% CI, 0.36-0.60]), and CRP-related DNAm (β, 0.55 [95% CI, 0.43-0.68]), with Black respondents experiencing faster epigenetic aging and greater CRP-related DNAm than White respondents. Conversely, Black respondents exhibited lower values than White respondents on TNF-α–related DNAm (β, −0.41 [95% CI, −0.55 to −0.27]).

**Figure 1.**
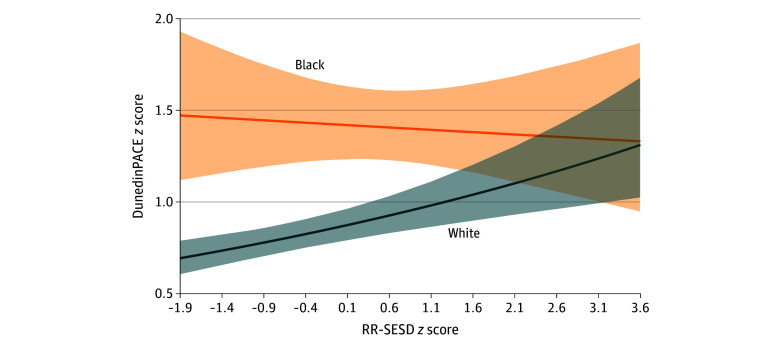
Line Graph of the Association Between Adolescent Exposure to Racism-Related Structural Economic and Social Disadvantage (RR-SESD) and Epigenetic Aging in Early Midlife by Race

**Figure 2.**
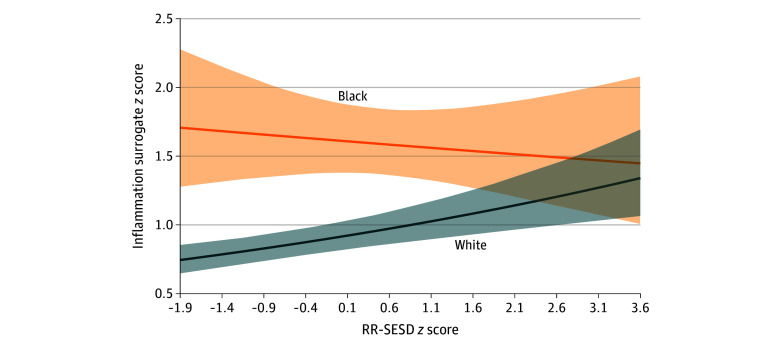
Line Graph of the Association Between Adolescent Exposure to Racism-Related Structural Economic and Social Disadvantage (RR-SESD) and C-Reactive Protein–Related DNA Methylation in Early Midlife by Race

Model 2 revealed differential associations by race for DunedinPACE (Table 2) and the CRP surrogate (Table 3). For both outcomes, the RR-SESD × race interaction term was significant and negative (DunedinPACE: β, −0.13 [95% CI, −0.25 to −0.02]; CRP-related DNAm: β, −0.14 [95% CI, −0.25 to −0.02]). Coupled with positive coefficients for RR-SESD and race, these findings suggest that while Black respondents experienced faster epigenetic aging and greater CRP-related DNAm than White respondents overall, the association between adolescent exposure to RR-SESD and these outcomes was slightly negative for Black respondents, yet positive for White respondents (Figure 1 and Figure 2). For example, White respondents living in counties at the third quartile of RR-SESD exposure experienced 7.32 (95% CI, 4.95-9.70) additional days of epigenetic aging acceleration (DunedinPACE) per year compared with White respondents from first-quartile counties, amounting to almost 0.40 years (or 146 days) of accelerated epigenetic aging over 20 years. In contrast, Black respondents from third-quartile counties exhibited 1.16 fewer days of epigenetic age acceleration compared with Black respondents from first-quartile counties, totaling almost 0.06 years (or 23 days) of slower epigenetic aging over 20 years. A similar pattern was observed for the CRP surrogate measure, with White respondents experiencing a more pronounced positive association between RR-SESD exposure and CRP-related DNAm than Black respondents.

## Discussion

This study used nationally representative survey and DNAm data to investigate the association between adolescent exposure to socioeconomic and segregation features of structural racism and epigenetic aging and inflammation-related DNAm in early midlife in the US. To our knowledge, this is the first study to prospectively assess early-life contexts of structural disadvantage and to examine its association with epigenetic processes 20 years later.

Our findings produced 2 major conclusions. First, exposure to RR-SESD in adolescence was associated with accelerated epigenetic aging and CRP-related DNAm by early midlife, even after adjusting for self-reported race and family socioeconomic status. These findings are consistent with previous work documenting significant impacts of early-life environments, including counties, on epigenetic and aging processes later in life.^52,53,54,55^ The organization of, and resources available within, counties are largely shaped by the historical and ongoing impacts of structural racism, which encompass centuries of social exclusion, violence, and resource misappropriation.^12,56^ Consequently, counties characterized by socioeconomic disadvantages and segregation may expose residents to more risk factors, while limiting access to health-relevant resources and opportunities. Experiencing these environments in early life may differentially place individuals on trajectories marked by accumulating advantages and/or disadvantages across the life course, ultimately influencing epigenetic markers of aging and inflammation by early midlife.

While the differences we observed may appear modest (eg, approximately 0.45 years of additional epigenetic aging acceleration [GrimAge2] for individuals who lived in counties at the third vs first quartile of the RR-SESD latent factor distribution), this difference amounts to approximately 9 years of accelerated aging across a 20-year span. Prior work has demonstrated that even small increases in epigenetic age acceleration are associated with elevated mortality risk and aging-related diseases.^57,58^ Detecting these differences in early midlife, before clinical manifestations of aging-related diseases typically appear, provides critical insight into the biological embedding of early social exposures and potential early warning signs of future disparities.

The second major conclusion is that the association between adolescent exposure to RR-SESD and epigenetic markers of aging and inflammation in early midlife differed by race. While Black respondents experienced faster epigenetic aging and greater CRP-related DNAm than White respondents, regardless of early-life context, they showed less steep or declining changes in epigenetic aging and CRP-related DNAm at higher levels of RR-SESD exposure. In fact, disparities in outcomes between Black and White respondents were smallest or nonsignificant among those who grew up in counties characterized by higher levels of socioeconomic disadvantage and segregation, and largest among those who grew up in counties marked by socioeconomic advantages and fewer proportions of Black residents (Figure 1 and Figure 2). Two countervailing mechanisms may explain these patterns. First, given historical and ongoing systemic barriers to socioeconomic resources and opportunities in the US, Black individuals have generally been restricted from geographic spaces containing health-promoting resources. Thus, unique and sustained efforts, stressors, and strains may be required and experienced among those able to access, remain in, and navigate socioeconomically advantaged counties.^59,60,61^ Such efforts, stressors, and strains may take a physiologic toll,^43,53,62^ resulting in faster epigenetic aging and inflammation-related DNAm among Black individuals in the US relative to their White counterparts. Simultaneously, there may be unique resources in areas that are considered disadvantaged. Prior work suggests that living in or navigating racially homogenous areas is related to better health outcomes—particularly by reducing race-related stress and improving feelings of belonging and support.^63,64^ These resources may help buffer the impacts of socioeconomic disadvantages and stressors on epigenetic and aging processes.

### Limitations

Several study limitations should be noted. This study considered one specific operationalization and dimension of structural racism at a specific geographic level. While RR-SESD captures a critical, theoretically grounded pathway linking racism to epigenetic aging, other indicators and dimensions of racism likely play an important role in shaping epigenetic processes. Future work should leverage opportunities to expand on these conceptualizations and explore manifestations of other subsystems involved in constituting racism at various geographic scales. Additionally, only Black and White respondents were considered, thus our findings may not be generalizable to other racial or ethnic groups in the US. Finally, we did not assess intermediate mechanisms linking adolescent exposure to structural racism and epigenetic processes in early midlife, which is beyond the scope of this study. Indeed, contemporaneous environments, which are likely correlated with adolescent exposures, may play a role in the observed associations. Future work should examine the role of various individual-level factors (eg, health behaviors, socioeconomic resources, and stressors) and contextual factors (eg, exposure to racial violence, air pollution, and health care access) across the life course as potential mediators.

## Conclusions

In this prospective cohort study of adults in early midlife in the US, we found that adolescent contexts are important factors for accelerated epigenetic aging and CRP-related DNAm. This association constitutes one pathway through which structural racism becomes biologically embodied. These results have important public health implications, suggesting that interventions targeting county-level socioeconomic and segregation factors—while also tailoring approaches to account for unique stressors and resources experienced by different racial groups across varying contexts—could mitigate disparities in aging-related diseases.

## Notes

Supplement 1.
eMethods

eReferences
**eTable 1.** Comparison of Analytic Sample With Add Health Wave V Biomarker and DNA Methylation Samples Across Key Covariates **eTable 2.** Unimputed Weighted Descriptive Statistics of Study Variables, Total and by Race

Supplement 2.
Data Sharing Statement
