# Supplementary material for: Structural Disadvantage in Adolescence and Biological Aging in Early Midlife
Source: JAMA Netw Open. 2026 May 11;9(5):e2611913. doi: 10.1001/jamanetworkopen.2026.11913 (PMC13162074; doi:10.1001/jamanetworkopen.2026.11913)
Supplement: Supplement 1. — eMethods eReferences eTable 1. Comparison of Analytic Sample With Add Health Wave V Biomarker and DNA Methylation Samples Across Key Covariates eTable 2. Unimputed Weighted Descriptive Statistics of Study Variables, Total and by Race [file jamanetwopen-e2611913-s001.pdf]

Supplementary Online Content

Hargrove TW, D'Alessio AS, Tuder S, et al. Structural disadvantage in adolescence and biological aging in early midlife. *JAMA Netw Open*. 2026;9(5):e2611913. doi:10.1001/jamanetworkopen.2026.11913

eMethods

eReferences

eTable 1. Comparison of Analytic Sample With Add Health Wave V Biomarker and DNA Methylation Samples Across Key Covariates

eTable 2. Unimputed Weighted Descriptive Statistics of Study Variables, Total and by Race

This supplementary material has been provided by the authors to give readers additional information about their work.

© 2026 Hargrove TW et al. *JAMA Network Open*.

eMethods

Detailed Description of Each Epigenetic Clock and Inflammation Surrogate

GrimAge Version 2[1] is based on 10 DNAm plasma proteins (including DNAm based surrogates for adrenomedullin levels, beta-2 microglobulin, cystatin C, growth differentiation factor 15, leptin, plasminogen activation inhibitor 1, tissue inhibitor metalloproteinase 1, smoking pack-years, C-reactive protein, and hemoglobin A1C), age, and sex. The clock is strongly associated with time to death, cancer, and coronary heart disease with high associations with markers of cardiovascular disease, obesity, type 2 diabetes, and inflammation. Evidence also suggests GrimAge Version 2 is more strongly associated with age-related phenotypes than GrimAge Version 1.

PhenoAge[2] is trained on 9 clinical biomarkers (including albumin, creatinine, serum glucose, C-reactive protein, lymphocyte percent, mean cell volume, red cell distribution width, alkaline phosphatase, and white blood cell count) and chronological age. The measure is based on weighted methylation values at 513 CpGs in whole blood. PhenoAge predicts all-cause mortality, cancer, physical function, and Alzheimer's disease.

DunedinPACE[3] is based on 173 CpG sites and identifies the rate of physiological decline for every 12 months of calendar time in the years prior to sample collection. Specifically, it tracks within-individual decline in 19 indicators of organ-system integrity, including cardiovascular, metabolic, renal, hepatic, immune, dental, and pulmonary systems, across four time points (ages 26, 32, 38, and 45). Prior work indicates that DunedinPACE provides a more precise measurement of the Pace of Aging relative to the DunedinPoAm, or the Dunedin Study Pace of Aging from methylation,[4] and is associated with morbidity, mortality, and disability.

C-reactive protein (CRP)-based DNAm surrogate[5] utilizes 218 CpGs identified in epigenome-wide association studies (EWAS) that are associated with serum CRP. We applied coefficients from meta-analyses of those EWAS studies as weights to the beta values of the CpGs, which are then summed to form the surrogate. The surrogate is adjusted for age, sex, white blood cell proportions, smoking, body mass index, and technical covariates relating to the implementation of the EWAS (control probes, chip, row, batch, etc.). It is associated with other inflammatory markers, lung function, chronic obstructive pulmonary disease, and some cardiometabolic diseases.

Circulating tumor necrosis factor-alpha (TNF-α)-based DNAm surrogate[6] is based on 8 CpG sites and is also adjusted for age, sex, white blood cell proportions, smoking, body mass index, and technical covariates from the EWAS (array, row, column). The surrogate is associated with risk of incident coronary heart disease. Similar to the CRP-surrogate, it uses CpG sites identified in EWAS studies and applies coefficients from meta-analyses of those studies as weights to the beta values of the CpGs, which are then summed to form the surrogate.

Additional Detail of Confirmatory Factor Analysis

Confirmatory factor analysis was conducted to derive a latent construct of exposure to county-level racism-related structural economic and social disadvantage in early life. Five indicators

© 2026 Hargrove TW et al. *JAMA Network Open*.

from the 1990 decennial census were used: 1) proportion of residents with income below the 1989 federal poverty level; 2) proportion of residents age 25 years or older with a Bachelor's degree or more; 3) proportion of residents over age 18 who are unemployed; 4) proportion of residents who identify as Black; and 5) proportion of county youths age 4-19 years living with a mother who did not graduate from high school, is divorced or separated, and is below the1989 poverty level. The factor analysis was completed at the county unit of analysis (n=267). The latent model has two degrees of freedom and is over-identified by the N_θ-Rule and the Factor Complexity of One with Correlated Errors Rule.[7,8]

Latent model equation:

Let Z_1 = Proportion of residents with income below poverty level; Z_2 = Proportion of county residents with a Bachelor's degree or more; Z_3 = Proportion of unemployed county residents; Z_4 = Proportion of county resident identifying as Black; Z_5 = Proportion of county youths with a mother who did not graduate from high school, is divorced or separated, and is below the1989 poverty level; L_1 = Racism-Related Structural Economic and Social Disadvantage (latent factor)

Then,

$$Z_1 = L_{1i} + \varepsilon_{1i}$$
$$Z_2 = \alpha_2 + \lambda_{21}L_{1i} + \varepsilon_{2i}$$
$$Z_3 = \alpha_3 + \lambda_{31}L_{1i} + \varepsilon_{3i}$$
$$Z_4 = \alpha_4 + \lambda_{41}L_{1i} + \varepsilon_{4i}$$
$$Z_5 = \alpha_5 + \lambda_{51}L_{1i} + \varepsilon_{5i}$$

Where $C(\varepsilon_{Z_1}, \varepsilon_{Z_4}) \neq 0$, $C(\varepsilon_{Z_1}, \varepsilon_{Z_5}) \neq 0$, $C(\varepsilon_{Z_4}, \varepsilon_{Z_5}) \neq 0$, all other ε_{zj} are uncorrelated with each other, $C\left(\varepsilon_{Z_j}, L_1\right) = 0$, $C(\varepsilon_{zj}, \varepsilon_{L_1}) = 0$, $E\left[\varepsilon_{Z_j}\right] = 0$ for all j=1,...,5 measurement variables and i = 1,…,267 counties.

Latent Variables:				
	Estimate	Std.Err	z-value	P(>|z|)
Latent factor				
Poverty	1	0.891	0.893	
Bachelor's degree	-0.765	0.065	-11.71	0
Unemployed	0.928	0.067	13.788	0
Prop. Black	0.371	0.08	4.659	0
At Risk Youth	0.723	0.061	11.91	0
Covariances:				
	Estimate	Std.Err	z-value	P(>|z|)
Poverty				
Prop. Black	0.273	0.05	5.513	0
At Risk Youth	0.221	0.05	4.396	0
Prop. Black				
At Risk Youth	0.618	0.075	8.25	0
Variances:				
	Estimate	Std.Err	z-value	P(>|z|)
Poverty	0.202	0.052	3.906	0

© 2026 Hargrove TW et al. *JAMA Network Open*.

Bachelor’s degree	0.531	0.061	8.72	0
Unemployed	0.313	0.051	6.179	0
Prop. Black	0.887	0.095	9.364	0
At Risk Youth	0.581	0.076	7.677	0
Latent factor	0.795	0.1	7.914	0
R-Square:				
	Estimate			
Poverty	0.798			
Bachelor’s degree	0.467			
Unemployed	0.686			
Prop. Black	0.11			
At Risk Youth	0.417			

Fit Statistics for CFA

	Standard	Scaled
Comparative Fit Index	0.994	0.994
Tucker-Lewis Index	0.969	0.972
Robust Comparative Fit Index		0.995
Robust Tucker-Lewis Index		0.973
RMSEA	0.107	0.085
Model Chi-Square Test Statistic	8.171	5.901
P-value	0.017	0.052

Note: We have a smaller sample size for this model, which led us to prioritize the Comparative Fit Index and Tucker-Lewis Index over the Model Chi-Square Test and RMSEA for determining goodness of fit.

Path diagram

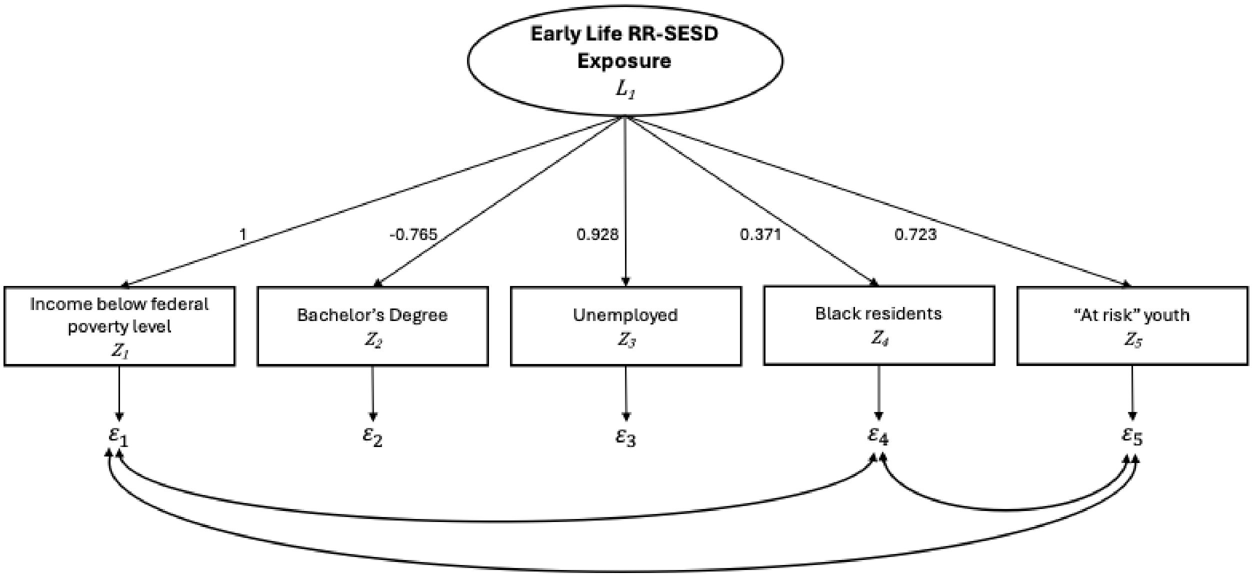

© 2026 Hargrove TW et al. *JAMA Network Open*.

eReferences

1. Lu AT, Binder AM, Zhang J, et al. DNA methylation GrimAge version 2. *Aging (Albany NY)*. 2022;14(23):9484
2. Levine ME, Lu AT, Quach A, et al. An epigenetic biomarker of aging for lifespan and healthspan. *Aging (Albany NY)*. 2018;10(4):573-591. doi:10.18632/aging.101414
3. Belsky DW, Caspi A, Corcoran DL, et al. DunedinPACE, a DNA methylation biomarker of the pace of aging. *eLife*. 2022.11:e73420. doi:10.7554/eLife.73420
4. Belsky DW, Caspi A, Arseneault L, et al. Quantification of the pace of biological aging in humans through a blood test, the DunedinPoAm DNA methylation algorithm. *Elife*. 2020;9:e54870.
5. Ligthart S, Marzi C, Aslibekyan S, et al. DNA methylation signatures of chronic low-grade inflammation are associated with complex diseases. *Genome Biol*. 2016;17(1):255. doi:10.1186/s13059-016-1119-5
6. Aslibekyan S, Agha G, Colicino E, et al. Association of Methylation Signals With Incident Coronary Heart Disease in an Epigenome-Wide Assessment of Circulating Tumor Necrosis Factor α. *JAMA Cardiol*. 2018;3(6):463-472.
7. Bollen KA. *Elements of Structural Equation Models (SEMs)*. Cambridge University Press; 2025.
8. Davis, W. R. (1993). The FC1 Rule of Identification for Confirmatory Factor Analysis: A General Sufficient Condition: A General Sufficient Condition. *Sociological Methods & Research*, *21*(4), 403-437. https://doi-org.libproxy.lib.unc.edu/10.1177/0049124193021004001

© 2026 Hargrove TW et al. *JAMA Network Open*.

eTable 1. Comparison of Analytic Sample With Add Health Wave V Biomarker and DNA Methylation Samples Across Key Covariates

	Wave V Biomarker Sample		Wave 5 DNAm Sample		Analytic Sample	
	N	%	N	%	N	%
Sample size	5381		4621		3788	
Race						
Black	1064	19.8%	885	19.2%	972	25.7%
White	3421	63.6%	2973	64.3%	2916	77.0%
Other	896	16.7%	763	16.5%	0	0.0%
Sex						
Male	2134	39.7%	1839	39.8%	1503	39.7%
Female	3247	60.3%	2782	60.2%	2285	60.3%
Wave I age (mean)	15.5		15.5		15.5	
Wave V self-rated health						
1	931	17.3%	807	17.5%	664	17.5%
2	1990	37.0%	1714	37.1%	1418	37.4%
3	1711	31.8%	1473	31.9%	1205	31.8%
4	607	11.3%	509	11.0%	409	10.8%
5	131	2.4%	113	2.4%	87	2.3%
Missing	11	0.2%	5	0.1%	5	0.1%
Wave V College Attainment						
College degree or more	2488	46.2%	2138	46.3%	1774	46.8%
Less than college	2882	53.6%	2478	53.6%	2009	53.0%
Missing	11	0.2%	5	0.1%	5	0.1%
Parent College Attainment				0.0%		
College degree or more	2150	40.0%	1859	40.2%	1586	41.9%
Less than college	3118	57.9%	2667	57.7%	2141	56.5%
Missing	113	2.1%	95	2.1%	61	1.6%
SR Factor (z-score) (mean)	-0.25		-0.25		-0.25	

© 2026 Hargrove TW et al. *JAMA Network Open*.

eTable 2. Unimputed Weighted Descriptive Statistics of Study Variables, Total and by Race

	Total	Black	White
Biological Aging Clocks			
PhenoAge	30.137<br>(.129)	30.118<br>(.335)	30.142<br>(.138)
GrimAge2	48.230<br>(.123)	49.785<br>(.268)	47.848<br>(.136)
DunedinPACE	1.020<br>(.003)	1.085<br>(.007)	1.004<br>(.003)
CRP Surrogate	.034<br>(.000)	.040<br>(.001)	.033<br>(.000)
TNF-α Surrogate	-.003<br>(.000)	-.006<br>(.001)	-.002<br>(.000)
RR-SESD Latent Factor	-.148<br>(.015)	.215<br>(.041)	-.237<br>(.017)
Age at Wave I	15.448<br>(.010)	15.676<br>(.096)	15.393<br>(.047)
Age at Wave 5	38.422<br>(.010)	38.736<br>(.096)	38.345<br>(.047)
Sex			
Female	.509<br>(.011)	.499<br>(.026)	.511<br>(.013)
Male	.491<br>(.011)	.500<br>(.026)	.489<br>(.013)
Parental Income	48.967<br>(1.169)	33.725<br>(1.886)	52.195<br>(1.343)
% missing	19.90%	28.10%	17.50%
Parent Educational Attainment			
Less than college	.619<br>(.011)	.707<br>(.022)	.598<br>(.012)
College or more	.381<br>(.011)	.293<br>(.022)	.402<br>(.012)
% missing	1.60%	2.10%	1.50%
Years in Residence	7.311<br>(.128)	5.873<br>(.284)	7.654<br>(.142)
% missing	0.9%	2.60%	0.40%
N	3,788	872	2916

Note: Means and standard errors are presented for biological aging clocks, structural racism latent factor, age, parental income, and years in recent residence. Proportions and standard errors are presented for sex and parental educational attainment. RR-SESD = Racism-Related Structural Economic and Social Disadvantage. Parental income is measured in $1,000's.

© 2026 Hargrove TW et al. *JAMA Network Open*.
