# Supplementary material for: Structural Disadvantage in Adolescence and Biological Aging in Early Midlife
Source: JAMA Netw Open. 2026 May 11;9(5):e2611913. doi: 10.1001/jamanetworkopen.2026.11913 (PMC13162074; doi:10.1001/jamanetworkopen.2026.11913)
Supplement: Supplement 2. — Data Sharing Statement [file jamanetwopen-e2611913-s002.pdf]

Data Sharing Statement

Hargrove. Structural Disadvantage in Adolescence and Biological Aging in Early Midlife. *JAMA Netw Open*. Published May 11, 2026. doi:10.1001/jamanetworkopen.2026.11913

Data

Data available: Yes
Data types: Deidentified participant data
How to access the data: Contact addhealth_srw_data@cpc.unc.edu
When available: Currently available

Additional Information

Who can access the data: Researchers who apply for a restricted-use contract with the National Study of Adolescent to Adult Health (Add Health)
Types of analyses: Any research-based analysis
